# CAN I ACCESS MY PERSONAL GENOME? THE CURRENT LEGAL POSITION IN THE UK

**DOI:** 10.1093/medlaw/fwt027

**Published:** 2013-10-17

**Authors:** Jane Kaye, Nadja Kanellopoulou, Naomi Hawkins, Heather Gowans, Liam Curren, Karen Melham

**Affiliations:** * Department of Population Health, University of Oxford, Oxford, UK; ** School of Law, University of Edinburgh, Edinburgh, UK; *** School of Law, University of Exeter, Exeter, UK; **** Research Services, University of Oxford, Oxford, UK

**Keywords:** bioethics, DNA sequencing, genetic research, genome, research ethics, research findings

## Abstract

This paper discusses the nature of genomic information, and the moral arguments in support of an individual's right to access it. It analyses the legal avenues an individual might take to access their sequence information. The authors describe the policy implications in this area and conclude that, for now, the law appears to strike an appropriate balance, but new policy will need to be developed to address this issue.

## I. INTRODUCTION

The Human Genome Project was a collaborative effort by many scientists and laboratories around the world, and resulted in the human genome being sequenced for the first time.^1^ Completed in 2001,^2^ this landmark scientific project has provided a unique reference library for the scientific community. It is estimated to have cost US$2.7 billion and involved 20 different laboratories around the world.^3^ As sequencing technology is improving, it is becoming less experimental and more routine. The result is that sequencing costs are falling and the possibility of a whole genome being sequenced for $1000 is getting closer.^4^ With the advances in next-generation sequencing technology, we are now entering a new phase where individual whole genome sequencing will no longer be the sole domain of specialist laboratories involving considerable effort, time, and expense. Whole genome sequencing is fast becoming a technology that will start to be used routinely by researchers and ultimately will be employed within the clinic to assist with personalised diagnosis and treatment strategies.^5^

Up until now, best practice has been that research participants were not given access to their sequence information produced through the research process. This approach is increasingly being questioned as sequencing costs fall and sequencing techniques are becoming more robust and richer in the amount of detail they can detect, which has implications for diagnosis and treatment. As research knowledge increases, the clinical utility of sequence information is also improving. There are a number of genes that indicate an elevated risk of serious treatable conditions, such as the gene for the low density lipoprotein receptor which indicates an increased risk of familial hypercholesterolaemia that can be lowered by taking statins.^6^ At the same time, the quality and quantity of the data continue to increase, making it increasingly difficult to anonymise sequence data, which is uniquely identifiable.^7^ Therefore, there is increased potential to reveal ‘incidental’ findings—those findings that were not anticipated at the start of the study^8^ or findings that have implications for individuals and their families that may have previously been unknown. A recent report by the American College of Medical Genetics (ACMG) recommends that a limited menu of incidental findings should be fed back to those who have had a genetic test in a clinical setting. These should be fed back routinely through the clinical setting but do not give the patient the opportunity to opt out of receiving the information.^9^ Having access to this personal information may enable participants to better understand their health conditions and make health-related decisions accordingly. The establishment of a number of direct-to-consumer testing companies^10^ that allow individuals access to their personal genome for a fee has also been a basis for questioning the policy of not giving individuals access to their personal genome. This had led to a heated debate within the bioethics and scientific community as to whether individuals should be entitled to their own sequence information generated through the research process.^11^ While there have been a number of papers that have advocated that there is a moral obligation to disclose relevant risks to individuals,^12^ there have not been any papers that have analysed whether individuals have a legal basis to access their own personal genome information. The purpose of this paper is to fill this gap in the literature by analysing the legal position in the UK as to whether individuals have a right to access their personal genome information (i.e. data rather than samples). We confine our main discussion to three heads of law that concern information—the Data Protection Act 1998 (DPA), the Freedom of Information Act 2000 (FOIA), and the Human Rights Act 1998 (HRA). It is worth noting that data protection law in the UK is likely to undergo substantial change over the foreseeable future, due to a review of the Data Protection Directive 95/46/EC.^13^ Currently in the UK, there is no legislation that focuses specifically on patients' rights, though at a European^14^ and international level^15^ such legal instruments exist. Therefore, these heads of law are the primary avenues through which an individual could base an action to obtain access to their personal genome data within the UK.

This paper will firstly discuss the nature of sequence information and how it is used in research, and then it will identify the type of research findings that might be of interest to individuals or their families based on the genome sequence. Secondly, it will discuss the moral arguments to support an individual's right to access four different types of research findings; these are raw sequence data, general research findings, patient specific findings, and patient-specific incidental findings. Thirdly, it will analyse the legal avenues open to individuals and whether it is possible for individuals to obtain access to the four specific types of information that are generated through the research process.^16^ Our conclusion is that there is currently no certain legal avenue for participants through these heads of law, despite the fact that there are compelling reasons why individuals might want to, and should have access to their genome sequence. The final section in this paper discusses the policy implications of these findings, suggesting that currently the law strikes the right balance regarding access to personal sequence information but that, as whole sequence information becomes more commonplace in healthcare, this position will need to be reviewed.

## II. THE NATURE OF GENOMIC INFORMATION

Genomic research is improving our understanding of how an individual's DNA inheritance may determine individual risk of disease or their response to or ability to metabolise drugs. The difference between whole genome sequencing and many of the sequencing techniques that are in common use is that whole genome sequencing provides information that could be used to determine an individual or familial risk for a number of conditions at the same time. The inherited nature of DNA means that information has implications not just for the individual but also for other family members.^17^ DNA can provide information that can be used for a number of different purposes such as health-related reasons, for determining family-lineage and ethnicity.^18^ Because of the sheer size of the genome and the information that it contains, previous analysis techniques have only been able to scan small segments of the genome on specific chromosomes. Therefore, until now the scientific enquiry has been focused upon specific genes and diseases. As the technology improves, understanding of the functionality of the genome will also increase. The broader scope afforded by improved technology will be able to encompass the relationship between different parts of the genome and investigate the functioning of whole cells. This in turn will influence the classification of diseases and potentially how healthcare is organised. As the knowledge about the significance of DNA for disease development improves, the potential for this information to become more relevant for healthcare will increase. Whole genome sequencing will therefore be able to be used to identify a range of conditions simultaneously not just for the individual but also for other family members.^19^

Another significant feature of genomic information is that it is uniquely identifiable to individuals: very small amounts of DNA may uniquely identify an individual.^20^ Medical researchers have traditionally utilised the twin pillars of consent and anonymisation to protect individuals involved in research. Current research governance requires the anonymisation of samples through the removal of personal identifiers when they are shared with other researchers. This means that the sequence data will not be immediately identifiable. However, if the sequence data are held by third party researchers with names and addresses, it will be possible to link the non-identifiable sequence data with the sequence data that have names and addresses attached. The nature of DNA is such that it may still be possible to select an individual from a group and identify them, even if the researchers remove all their ‘external’ identifiers. In the past, aggregated genomic information was publicly released, but this practice ceased after researchers demonstrated that, when a reference sample is available, it is possible to identify that individual out of a large aggregated sample.^21^

When whole genome sequencing is more commonplace, therefore, it will also be possible to track the sequence data back through the research pathway just by looking at the sequence data alone. This means that it would not be difficult for a researcher or institution to distinguish individual sequence data and possibly link it to other personally identifiable information even if the identifiers had been removed. Currently, the systems in place are not designed to track individuals: management pathways would have to be established to enable this to happen if individuals did want access to their personal genome. However, in the future where whole genome sequencing becomes commonplace, lack of tracking capability may no longer be the norm. Already, whole genome or exome sequencing is being planned to occur more routinely in non-anonymised cohorts, which will make the linking straightforward.^22^ This means that arguments against disclosure of personal information to participants on the basis that participants are not identifiable are less robust.

## III. WHAT TYPES OF INFORMATION MIGHT PARTICIPANTS ACCESS?

Information which is generated in the course of research does not meet the same standards of quality control that are required for clinical care. In the research context, to obtain sequence information, researchers must first obtain a biological sample (usually blood or saliva, but sometimes other types of tissue may be used). Questions of access by individuals to physical samples are regulated by the statutory scheme in the Human Tissue Act (HTA). Although the HTA regulates access to physical samples, it does not regulate the ways in which research participants might obtain access to information, and therefore the HTA is outside the scope of this paper, where we focus on data.^23^ Information that is generated within a research context is very different from information which is obtained through diagnosis and treatment and is required for an individual's clinical care. In the research context, the focus is on a research question, in the first instance on the treatment of particular individuals, findings may not have clinical significance for research participants, and research sample handling systems do not have to be as rigorous as is the case in clinical work. Information from a clinical diagnosis or intervention would be entered in a medical record. Patients can obtain access to this information by using their rights under various legal avenues.^24^ The information generated through research does not necessarily find its way into a person's medical record and therefore cannot be routinely accessed. Therefore, it is important to understand whether there is a legal basis for a participant to access personal sequence information.

There are different types of information which might be generated from genomic research and which may be of interest to individuals. We have identified four different types of information that fall under the description of ‘genomic information’ which would be created through the various stages of the research process. Each of these types of genomic information is subject to different types of legal controls or obligations depending on whether they can be linked to an identifiable living individual.

### A. Raw Data

This consists of the SNP information,^25^ or genetic sequence information (either complete, or exome sequence information),^26^ and can be represented as a read out of the base pair sequence^27^ on an individual's genome. Although the vast majority of the general public (and indeed many genomic scientists) would be unable to interpret this information to provide any meaningful conclusions, online applications^28^ (such as SNPedia and Promethease) encourage members of the public to share their genomic information in an open source manner, and also provide tools to conduct genomic analysis. It is likely that such applications will become increasingly sophisticated in the next few years and some medical practitioners may be able to draw meaningful conclusions from the information. This type of information could be obtained by providing a sample to a company which undertake either GWAS sequencing (such as 23 and Me) or a company that carries out whole genome sequencing.

### B. General Research Findings

These are the findings obtained from the analysis of the study and are generally provided through the scientific publications, and participant newsletters and websites. The information is about the results of the study as a whole and does not provide information at a patient specific level.

### C. Participant-Specific Research Findings

These are the results of the particular study that have relevance to an individual patient. These have been described as ‘pertinent’ findings in the UK10K study^29^ as they may be findings that relate to the type of study that has been carried out, for example, finding a particular genetic variant postulated to cause diabetes, in a patient taking part in a diabetes study. Some research leads to information which is relevant on a population level—and there may be relevant findings for a small number of participants on an individual level—but research results may need to be verified by further research. It is therefore not always clear at scientific level what this type of feedback might involve, or what the findings might mean to an individual.

### D. Participant-Specific Incidental Findings

This type of information refers to participant-specific findings that are not directly related to the purpose of the study. Wolf *et al*. define incidental findings as ‘a finding concerning an individual research participant that has potential health or reproductive importance and is discovered in the course of conducting research but is beyond the aims of the study’.^30^ For the purposes of this paper however, we wish to take a broader perspective. Wolf's definition limits incidental findings to those which might have health or reproductive importance. Such information would include clinically validated health findings such as a validated disease causing mutation, or a pharmacogenetic variant, and is clearly relevant information for the purpose of this paper. Incidental findings might be findings that have already been validated by other research and in some cases have already been used within a clinical setting. However, genomics research may also uncover other information about a participant which may be of interest to them, but which is not relevant to health or reproduction. The other type of finding relates to ‘non-health’ issues such as ethnicity and non-paternity.^31^ We will include this latter type of information in our definition of incidental findings.

## IV. SHOULD INDIVIDUALS BE GIVEN ACCESS TO RESEARCH INFORMATION?

The question of whether, and how, individuals can access information that comes to light in the course of research goes to the heart of the researcher–participant relationship; an understanding that needs to evolve in-line with changing scientific developments and social and cultural norms. The ethics of research involving human participants rests on the value of respect for individuals, their dignity, and bodily integrity. This value is enacted in the process of informed consent, which underscores voluntariness and includes the ability to withdraw from research.^32^ There is more to the participant–researcher research relationship, however, than a simple willingness of one individual to undergo an intervention for the benefit of another's research pursuit. This is evident in the movement within research and research ethics from an understanding of participants solely as donors—providing bodily material for a researcher's use with no further link between the parties—to an understanding of research as a social enterprise in which researchers and participants are both variously engaged. Such a development moves the researcher–participant encounter from an episode to a relationship within the social endeavour of translational research. It also opens more widely consideration of what parties owe to one another and the shared project, and on what grounds these obligations are based. Full consideration of the nature of this relationship is beyond the scope of this paper. In the current context, we consider how traditional biomedical principles of beneficence, non-malficence, autonomy, and justice^33^ may be employed in exploring participant access to research-generated genomic information. Much of the bioethics literature to date has focussed on the researchers' ethical obligations to disclose research data rather than the individual's right of access.^34^ In considering the respective ethical rights and obligations on individual access to one's genome, the literature emphasises the researcher's ‘offer’ to disclose research findings to participants and is largely silent on the participant's claim to access genomic information about themselves that is held by researchers. The claim for access therefore is traditionally seen as a passive claim vis-à-vis the researcher's (active) duty.^35^ From the researcher's perspective, arguments against disclosure have often focused on the distinction between care and research and have centred on concerns over the therapeutic misconception and diagnostic misperception, occurrence of harm, the individual's right not to know, and whether high cost and time-intensive burdens of disclosure could be detrimental for individuals and for research.^36^ The focus has been on non-maleficence. There has been a shift towards a more positive approach to notifying individuals of participant-specific research findings, extending as far as discussion of the obligations to look for moral obligations to do so.^37^ Arguments have been made to support the disclosure of research results to participants which include respect for individual autonomy and self-determination, the ethical prerogative to not regard the individual as a means to an end, considerations of reciprocity, and furthering intelligent communication between researchers and participants, which can foster trust and on-going public involvement in research.^38^ The arguments for allowing access by participants in the case of our four types of information are as follows.

### A. Raw Data and General Research Findings

General research findings are often distributed to research participants and the wider public through newsletters and websites as well as being made available through publications. Returning such findings to individuals is increasingly becoming part of common practice and does not raise the same ethical questions as allowing access to raw sequence data which relates to a specific individual. In the case of raw sequence data, there are a number of reasons why individuals might not be given access. The first is that the sequence data developed through the research process is not at the current time robust enough to be clinically useful. It would require a significant change in practice, protocols, and procedures to generate sequence information of a sufficient standard that could be given to individuals to form a basis for clinical decision-making. To do so, would involve considerable time and money. These are often the grounds on which feedback is refused.^39^ The principle at work is one of distributive justice, seeking to balance the request of participants with the resource priorities of often publicly funded research. If individuals really want access to this information, they could obtain it through other means such as a direct-to-consumer genetic testing company which through the paid contractual nature of the arrangement, would also take on the liability for the quality of the information.

On the other hand, there are strong intuitive counter-arguments that a whole genome sequence is a unique identifier and should be known by the person to whom it relates. This would recognise that genomic information is unique to the individual and so providing access to it could foster trust and on-going public involvement in research as this would be in the spirit of benefit sharing. These are often the motivations for providing research information through newsletters or websites. As genetic tests become more common, the desire for information and the expectations of research participants for receiving related results are on the increase.^40^ These more recent practices also form part of a broader cultural change where individuals are encouraged and become accustomed to actively seek—and also demand—more information about themselves.^41^ To the extent that this information is related to their health and living habits, it can ultimately lead to changes in how individuals perceive themselves and take care of themselves and their relatives. These considerations also find resonance in recent UK policy recommendations to support individual ‘responsibilisation’ in healthcare.^42^ There is increasing support that individuals should have access to their own sequence data simply because it is ‘theirs’, even though the utility of this information is still not fully understood. This recognises the autonomy of individuals. However, we consider that any interpretation of sequence information that has clinical significance has to be returned through a clinical context. This does not rule out the possibility of returning an individual's raw sequence data to them personally if it were thought appropriate. Rather it is a recognition that part of respect and dignity is the provision of material in a form in which it can be meaningful.

### B. Participant-Specific Research Findings and Incidental Findings

In the case of patient-specific findings, a number of recent studies examined participants' views about access to information results in genomic biobanking, which reveal extensive support for knowing individual research findings.^43^ There are several reasons why participants want to know this information, which range from health-related reasons to a curiosity interest, to family-lineage and related expectations, including self-discovery about one's ethnicity or history, linked to tales of personal as well as community identity and knowledge.^44^ This information could have significant implications for people's health that could help them avoid life-threatening conditions but also identify drugs that might be more suited to their metabolism using a personalised medicine approach. Access to this personal information may be crucial in enabling participants to have a better understanding of their health conditions and make health-related decisions accordingly**.** Genetic information is about identity as well as health. Recognising this and sharing information that may not seem clinically relevant but may be of import to a participant recognises that the interests of participants extend beyond those of research. Further, because it is a unique identifier, an individual's genetic sequence is intensely personal. Individual dignity or respect for persons can be recognised and supported through the provision of information that provides non-clinical meaning related to identity and familial relationships. Dignity can also be seen as a form of informational integrity: the privacy of individuals vis-a-vis such deeply personal information is equally important and places a duty on researchers to develop robust systems for information security. Where the old guarantees of anonymity are no longer possible, it is necessary to find other ways to safeguard these same values. Disclosing research results to participants adheres to the principle of respect of persons and the notion that individuals have the right, as self-determining agents, to receive research results if they so choose.^45^ Providing such results could encourage greater awareness of the benefits of research and in doing so would lead to greater public support for biomedical research.^46^ There is a danger that if serious treatable conditions are not reported back to individuals that this would affect public opinion negatively towards research.^47^ It would be considered morally reprehensible for a researcher to know that an individual had a serious treatable condition but to decide not to alert the participant or their treating physician. This could have a detrimental effect on the public's trust and support of researchers, and it could be argued that giving participants access to such information has the potential to place the relationship between researchers and research participants on more equal terms.^48^ Various approaches are proposed to give greater consideration to research participants' views and for research participants to express their preferences on the types of information they wish to receive through choosing various notification options of research results, usually at the moment of enrolment but also beyond.^49^ Such approaches would also support a participants' desire not to know certain findings or the right not to know. There could be negative effects on individuals if they do receive such information which cannot be acted upon. This may increase anxiety and so information should only be fed back if there are immediate clinical benefits.

From the participant's perspective, there are a number of compelling reasons why they might want to access personal genome information. As research knowledge becomes more available to the general public because of open access policies and as the implication of the genome on disease susceptibility and drug metabolism is better understood, this information will be significant and more accessible to the general public. Therefore, it would be unethical not to provide access to such information that was held by researchers, if it was in a form that was useful for clinical care, and if individuals wanted to have access to their specific information. While these arguments are compelling in terms of respect for persons and autonomy, they would require a significant change in the way that research is carried out and healthcare is delivered, but also in terms of current law surrounding access to personal information in the UK.

## V. THE LEGAL FRAMEWORK

The Oviedo Convention of 1997 (on Human Rights and Biomedicine),^50^ and its Additional Protocol of 2008 (concerning Genetic Testing for Health Purposes)^51^ assert that individuals are entitled to know any information collected about their health,^52^ which may be derived from a genetic test.^53^ It should also be noted that both the Convention and Additional Protocol qualify these provisions with reference to an individual's rights *not* to know about, or have access to this type of information. As significant and wide-reaching as these European rights of access to health information appear, they have no legal force in the UK beyond mere persuasion, as neither the Convention nor Additional Protocol have been signed or ratified. To assert similar rights of access in the UK, individuals must instead rely on specific national legislation, namely the Data Protection Act 1998; the Freedom of Information Act 2000; and the Human Rights Act 1998.

All of these statutes in principle uphold a right of access, but they are all subject to a variety of qualifications. Before analysing them in more detail, it is worthwhile considering the wider culture in the UK as regards access to medical information more generally. In the healthcare setting—indeed enshrined within the NHS Constitution—patients have a right of access to their own health records.^54^ The statutory basis for this right is found primarily in data protection law, but other acts come into play in certain scenarios.^55^ When it comes to research, it would seem that the default position is for participants not to be given access to individual-level genomic information largely because the quality of the sequencing and the fact that results may not have clinical validity or utility. This position, which clearly differs from the situation in the provision of healthcare, appears to have been adopted in an attempt to avoid complicating the research process, and overburdening researchers with non-research tasks. But to what extent would the law in the UK—namely the three specific Acts referred to above—support a research participant seeking to gain access to such information?

### A. Data Protection Act 1998

The Data Protection Act 1998 (DPA)^56^ is the keystone in UK information law, and it regulates the use of personal data held manually and on computer. The aim of the Act is to protect the rights of individuals (‘the data subject’) about whom data are obtained, stored, processed, or supplied.

The DPA only applies to data that have not been successfully anonymised. In order for the DPA to apply, the ‘personal data’ must relate to a living individual who can be identified from those data, or from those data and other information in the data controller's possession. The question of whether the data are anonymised depends to a great extent upon the security and non-disclosure safeguards that are put in place, such as removing identifiers, coding, firewalls, and aggregating data sets. In many cases, researchers involved in genomic research use systems such as coding (codes held by third parties) to anonymise the data to protect the privacy of research participants and individual researchers are not in a position to identify a research participant. It is therefore unclear as to whether the research results constitute anonymised ‘personal data’ for the purposes of the DPA. If data can be successfully anonymised, then the DPA does not apply. If the data used in genomic research is not truly anonymised, then it will fall within the ambit of the DPA, provided that such data also constitute ‘personal data’ for the purposes of the DPA. As technology improves and whole genome sequencing (at high resolution) becomes increasingly common, de-identification of data will become more difficult. Although sequencing technology and coding techniques may still mean that DNA itself does not constitute ‘personal data’, for the rest of this DPA analysis, the assumption is made that genomic research data can be regarded as personal data.

Personal data must be processed in accordance with the rights of data subjects provided under the DPA.^57^ Although section 7 DPA gives data subjects a right of access to a copy of the information comprising their personal data and a description of the personal data (subject to a small administrative charge), the rights of access are circumscribed by the so-called research exemption. The research exemption in section 33 DPA applies where the following relevant conditions are met:

1. the data are not processed to support measures or decisions with respect to particular individuals, and
2. the data are not processed in such a way that substantial damage or substantial distress is, or is likely to be, caused to any data subject.

Where section 33 applies, subject access does not have to be given if the data are processed in compliance with the relevant conditions, and the results of the research or any resulting statistics are not made available in a form which identifies data subjects.^58^ Assuming that genomic researchers do not deviate from standard ethical practice, section 33 should severely limit the use of section 7 for genomic research feedback requests. The exemption will apply in most cases in which requests for access to a research participant's genomic information is made.

Participants face another small hurdle in section 7(4) DPA, which states that complying with an access request is not required if to do so would mean disclosing information about another individual who can be identified from that information, except where the other individual has consented to the disclosure, or it is reasonable in all the circumstances to comply with the request without that individual's consent. If the analysis of the participant's genome depends on a comparison with another person's data, then it could be argued that subsection (4) is applicable. The reasonableness test in subsection (6) does not explicitly look at the requesting participant's need for access to the information, but rather considers the relationship between the researcher and the other individual.

There is a final hurdle in section 8 DPA, which provides that a copy of the information in permanent form does not have to be supplied if the supply of such a copy is not possible or would involve disproportionate effort. The effort and cost needed to extract and supply individual information from genomic research could be considered disproportionate. The Information Commissioner's Office emphasises that a narrow interpretation of ‘supply’ should be made (i.e. that it should be restricted to the actual act of communicating the information), and it stresses that the provision should only be used in exceptional cases;^59^ so this provision may not present much of an obstacle to a requesting participant.

This leads to the conclusion that the DPA is unlikely to provide genomic research participants with a right to access their genomic information. Even if DNA is determined to be personal data (which is unlikely until advances in technology and sequence sharing are made), the exemptions to the rights of access are such that refusal to grant access will be acceptable. There are good reasons why researchers and their institutions might be reluctant to provide this information to individuals, as to do so would require the development of new management pathways. However, the proposed changes to European data protection law would include ‘genetic data’ as personal information and therefore all of these different types of genetic information may in the future be considered as personal information.^60^

### B. Freedom of Information Act 2000

The Freedom of Information Act 2000 (FOIA) establishes the legal framework for access to information held by public authorities. An individual has the right to request information held by a public sector organisation under the FOIA, which became law in the UK in January 2005. The FOIA creates a statutory right of access and provides an extensive scheme for making information publicly available, which covers a wide range of public authorities, such as the NHS, local government, and education.

The FOIA applies to information held by public authorities and information held by other persons on their behalf. The FOIA will only be useful to research participants if it is determined that DNA itself is not personal data. Currently, we do not have any case law or decision on this specific point. Section 40 FOIA provides that if the information requested is the participant's personal data, then the DPA applies, and the FOIA does not, in which case only the DPA analysis above is then relevant. If it is another person's personal data, then it must comply with the data protection principles set out in the DPA, and disclosure of the information must not cause damage or distress (unless the public interest in disclosure outweighs the public interest in keeping the exemption—the public interest test). The case of *Common Services Agency v Scottish Information Commissioner* (*Scotland*)^61^ demonstrates how the FOIA and the DPA work together:^62^ if the data can be sufficiently anonymised, their disclosure falls under the standard FOIA provisions; if they cannot be, it has to comply with the DPA principles.^63,64^

The FOIA further provides that disclosing the information must not be a breach of confidence to a third party,^65^ and the information should not constitute a trade secret or prejudice the commercial interests of any person.^66^ The breach of confidence exemption would severely limit a research participant's right of access to other people's data under the FOIA, as it is difficult to imagine an example of medical research in which divulging another person's personal data without consent would not be a breach of confidence.^67^ Furthermore, given the commercial value of some research, perhaps the exemption will regularly be engaged.^68^

Perhaps the most relevant exemption is that regarding information intended for future publication contained in section 22 FOIA. For it to apply, at the time the request is made, the public authority must actually have an intention to publish the information (or someone else must have an intention to publish it),^69^ and, if it does, it must be reasonable in the circumstances that the information should be withheld from disclosure until the publishing date. It must also satisfy the public interest test (whereby the public interest in disclosure must outweigh the public interest in keeping the exemption). The underlying thinking behind the exemption is that, if the applicant will be able to obtain the information by another means, there is no need for a separate statutory route, and so the information must be published at some point in the near future, or a refusal to provide will probably be deemed to be unreasonable. Perhaps the fact that the information will be used for research and all the associated reasons against disclosing research findings early, for example a reduction in academic incentive,^70^ will help an authority to argue that its refusal to disclose before the publication date is reasonable and in the public interest.^71^

In addition, section 21 FOIA provides that: ‘Information which is reasonably accessible to the applicant otherwise than under section 1 is exempt information.’ As obtaining one's genome sequence becomes cheaper and easier, it may seem that this is more likely to be satisfied if a research participant is simply asking for his or her sequence; it must be remembered, however, that the FOIA deals with information—the specific genomic information that researchers hold about their participants will not be available from anyone else. Conclusively, the ICO's guidance on section 21 suggests that, for the information to be reasonably accessible, charges can only be made if a statutory scheme provides for a fee-charging information provider, or if the information is provided under the researching authority's publication scheme;^72^ neither of these cover where private genome sequence providers charge for their services.

The FOIA will, in general, not be useful to research participants, particularly because of the section 22 exemption and the fact that it cannot be used to access personal data. If a research participant wants general information about the research, this may be possible, but this would be unlikely in most genomic projects where there is an intention to publish the data at a later date, and a refusal to divulge the data before this date is reasonable.

### C. Human Rights Act 1998

We have seen how data protection and freedom of information laws—areas of the law whose very existence is rooted in human rights—can be used as the basis for a claim for access to information in specific circumstances, but what of a claim based on a more general application of an individual's rights under the European Convention on Human Rights and Fundamental Freedoms (the Convention)?^73^

Since the Human Rights Act 1998 (HRA) came into force in 2000, UK law must be interpreted and applied by courts in a manner compatible with the Convention. The decade of case law that has resulted dwells on two broad categories of dispute: firstly, those where a ‘public authority’^74^ (or some other legal entity ‘whose functions are of a public nature’^75^) are alleged to be acting in contravention of the Convention; and, secondly, those where Convention rights are sought to be applied in ways that create legal obligations on private individuals. In short, there are public and private dimensions to human rights law, with the latter being confusing territory for individuals to bring claims in. Before considering such claims in more detail, it is worth stating that there would, inevitably, be some similarities between a ‘pure’ human rights claim and those made under the more specific legislation already discussed, and it is perhaps unlikely that a claimant in a UK court would seek only to arm themselves with the Convention as a means of getting access to genomic information. Nonetheless, an examination of how certain Convention rights could bolster such a claim is a useful exercise.

Given the public/private dimension of human rights cases, the founding of any claim would turn on who and what the defendant is, and upon what basis they seek to deny the claimant access to information. If the defendant in question were a public authority or similar entity—for example the NHS, universities undertaking publicly funded work/functions of a public nature, or indeed the UK Biobank^76^—then the standing of a claim would be on firm ground. Seeking redress against a private defendant, for example a direct-to-consumer genetic test provider, with no obvious public function (and no doubt further supported, contractually, by its own standard terms and conditions) would still be possible. As the European Court of Human Rights has made clear, while the essential object of the autonomy-friendly Article 8 is to protect the individual against arbitrary interference by the public authorities, it does not merely compel the state to abstain from such interference: in addition to this negative undertaking, there may be positive obligations inherent in effective respect for private or family life.^77^ These obligations may involve the adoption of measures designed to secure respect for private life even in the sphere of the relations of individuals between themselves. This was most recently emphasised in *Mosley v United Kingdom*^78^ and *Von Hannover (No.2) v Germany*.^79^ This approach is also consistent with the Court's emphasis that the Convention is dynamic, reflective of social changes and ‘a living instrument which should be interpreted according to present-day conditions’.^80^

Article 8 is a qualified right and its application could be considered as a two-stage test: firstly, assess whether the rights set out in Article 8(1) are ‘engaged’; secondly, if Article 8(1) is engaged, determine whether any of the exceptions in Article 8(2)—which include the protection of health—override application of the rights. The reasons for a claim will therefore be relevant: is the claimant merely curious as to the information about him held by others, or are there other genuine concerns such as health matters, paternity, or an interest in ancestry behind a request? There is, of course, a qualified right of access to personal data under the DPA that would seem to permit genuine curiosity-based applications. Article 8 could be cited as giving further support to such applications, but in reality such applications will only be data protection claims. It is perhaps because of the particular nature of genomic information—whether it is considered ‘health’ or ‘medical’ in nature^81^—that Article 8 could provide some added weight to a claim for access, and there is judicial authority to back this up, as discussed below.

Before turning to rights of access per se, it is worth considering the more general application of Article 8 of the Convention to genetic information. In 2004, the House of Lords, when questioning the retention practices of DNA samples by the police in the *Marper* case,^82^ held that such practices did not engage Article 8(1), and in any event were justified by Article 8(2). A dissenting opinion of Baroness Hale, which was endorsed when the case was heard by the European Court of Human Rights in 2008,^83^ was notable for its observation that ‘there can be little, if anything, more private to the individual than the knowledge of his genetic make-up’.^84^ In light of this judgment in Strasbourg, the original *Marper* decision was deemed unlawful by the UK Supreme Court in 2011.^85^ There is additional authority from Strasbourg that Article 8(1) is engaged by the systematic retention of DNA samples, with justification founded upon the use which such samples ‘could conceivably be put in the future’.^86^

The European Court of Human Rights in the case of *K.H. v Slovakia*^87^ affirmed that an individual's right of effective access to personal data—without the need for justification—engages Article 8. Here requests made by several women, all of whom suspected they had been sterilised without their permission, for copies of data concerning their fertility were denied by a Slovakian public authority. The court held that the failure by the authority to uphold this right of access, without compelling reasons, constituted a breach of Article 8: in other words, the burden of proof should rest with the public authority to justify a refusal of access. While this ruling suggests that a public authority must, in general, supply medical records, it should be borne in mind that the court emphasised that the women's ‘moral and physical integrity’ was particularly relevant in this case, quite possibly due to the original lack of consent. In the less extreme case of participants in genomic research, who will have been required to sign an informed consent form prior to their involvement, a similar application of Article 8 cannot be guaranteed, particularly given the qualified nature of existing access rights under data protection law in the context of research. Another more recent case, *Gillberg v Sweden*,^88^ though not addressing the rights of access for research participants *per se*, makes it clear that assurances of confidentiality as between researcher and research participant will never be absolute.^89^ In this case, K and E, a sociologist and paediatrician, were granted access to confidential information by the Administrative Court of Appeal on certain conditions because they had shown a legitimate interest in the material in question.^90^ Their familiarity with the handling of confidential data, as well as the importance of independent validation of the methods used in research also weighed heavily in the Court's decision to allow access.^91^ This suggests that confidentiality may be trumped in certain situations where access to health information such as genomic information is required, for example, for further research use. Most pertinently, the court held that strict legal foundations of confidentiality (the Swedish Secrecy Act in this case) should take precedence over claims made in consent forms, and international instruments such as the Helsinki Declaration. While *Gillberg* addressed requests for access by researchers, it can serve as a reminder of the balance between confidentiality and access requests by genomic research participants, given the potential for genomic information to reveal confidential information about others. However, some commentators have argued that the case of *Gillberg* suggests that the Strasbourg courts might be moving towards a right of access.^92^

## VI. LEGAL RIGHTS OF ACCESS TO THE FOUR TYPES OF GENOMIC INFORMATION

Our classification of genomic information comprises four categories that could conceivably be generated in most biomedical research: raw data; general research findings; patient specific research findings; and patient-specific incidental findings. In all cases, it is likely that proper application of the FOIA and DPA will, on the assumption that both these Acts are compliant with the Convention, severely limit the scope for human rights-based claims of access.

(i) Raw data (which consists of the SNP information, or a complete genetic sequence, and can be represented as a read out of the base pair sequence on an individual's genome): These data stand a chance of being classed as personal data under present legislation,^93^ and thus caught by the subject access right of the DPA. Unlikely as this may be, the research exemption in the DPA would certainly present the researcher with a very strong basis upon which to resist such an application. The quality of raw data—which constitutes reams of many thousands of base pairs—is such that decisions about individuals using these data would be all but impossible: this would be the deciding factor in the successful application of the first part of the research exemption.^94^ If not personal data, there could be scope for a request under the FOIA if the research was conducted by a public authority^95^though it is very likely that such an application would be defeated on the grounds that the same information would, ultimately, be published in line with standard academic practices.^96,97^

(ii) General research findings (which are findings obtained from the analysis of the study): This information will not be personal data under the DPA, and accordingly the right of subject access will not materialise. Similarly, the lack of a direct link to an individual will doubtless prevent application of Article 8, or Article 2, of the Convention. As with raw data, a request to a public authority under the FOIA will again be subject to the publication exemption.

(iii) Patient-specific research findings (which are results of the particular study which have relevance to an individual patient): Clearly, this type of information stands a reasonable chance of being personal data, even if it is of a speculative nature. If sufficiently personal, getting the research exemption to apply to this category of genomic information may not be quite as straightforward as in the case of raw data. The ability of a researcher to deny access based on this exemption will hang on the significance of the data to the individual—if the data are so serious that any processing of them (without knowledge of the participant, it is assumed) constituted life or death matters, then the exemption is less likely to apply. Such considerations will, most likely, require further research to ascertain the significance of the information; if this is the case, then the exemption is likely to apply. FOIA considerations will be the same as the previous two categories.

(iv) Patient-specific incidental findings (which are not directly related to the purpose of the study): This information stands the greatest chance of being personal data, and therefore it may be difficult to use the research exemption as the basis for rejecting a subject access request. We say this because a researcher processing this type of information, particularly if it relates to health issues will have had to depart from the stated objectives of the research exemption that the information in question will not be of real significance to an individual, or individuals. The difficulty here is that paternity and ancestry information as well as health information could be found out from an analysis of sequence data and may also be of significance to the individual. This could be of interest to research participants and of real significance, but may not be gleaned from all research analyses. The more specific these enquiries become, the more likely the data are personal, and the less likely that both limbs of the research exemption will apply. In the unlikely event that the findings were not considered personal, then application to a qualifying researcher under the FOIA would again meet resistance through the publication exemption. This is based on the assumption that the researchers would still pursue publication of such information, even though it was not part of the original research plan.

## VII. CONCLUSIONS

It is evident from our analysis that participants in genomics research under current UK law have very limited rights to access the four types of information identified in this paper. However, the proposed changes to European data protection law may have implications for an individual's right of access to personal genetic information. At this time, it is not clear what the new European regulations will contain. Currently, individuals would find it difficult to establish a right of access in the case of raw sequence data and general research findings generated through the research process, but this is less certain in the case of patient specific research findings and patient-specific incidental findings. It is the individual-level data, which have implications for individuals that present the most challenges in terms of a legal right of access to this information by individuals. The strongest claim for access that could be made would be in relation to information with specific relevance to health and reproduction (or arguably paternity or ancestry), but even so, through the current legal avenues that exist in the UK it would be difficult for an individual to access data generated through the research process.

At the present time, the law strikes an appropriate balance in its attitude to the provision of genomic information, and that these limited rights of access are broadly appropriate, for two reasons. Firstly, at present, the information generated in the four categories identified is of limited significance and use to individuals in contrast to more defined and specific medical information, which is available through the medical record. However, as medical knowledge and technology develops, more situations may arise where there is a legitimate claim to access genomic information, particularly where this has direct medical relevance to the patient and has clinical utility. There is an impetus towards revealing more information to patients and participants, demonstrated by the ACMG report on incidental findings.^98^ These guidelines will have an international effect, and although they were targeted at clinical care rather than research, they challenge our perception of what information should be fed back. While the law in this rapidly evolving area may be adequate for now, it is unlikely to stay so for long. However, to make research data available to participants requires that the interface between clinical care and research be more closely aligned. This information should be in the medical record and then a patient should have an almost unfettered right of access, but currently research results are not always recorded in this way.

Secondly, there needs to be a consideration of the costs involved in providing access to such information for research participants. To develop appropriate management pathways will involve the allocation and expenditure of resources as well as the development of appropriate expertise. A clear policy decision would have to be made if such costs were to be borne by the research enterprise. For example, systems would need to be in place to ensure that there are appropriate quality controls so that information from the research context can be utilised in the clinic. These translational research models are starting to become more common in the UK and provide a way for information to be used and returned to individuals in an ethical manner. However, the research and clinical domains are regulated in very different ways and to ask researchers to provide feedback on individual findings would be inappropriate without the development of appropriate management pathways that only currently exist for clinically based research.

The use of whole genome sequencing is becoming more routine within research with more projects^99^ having the expertise and the funding to be able to carry out this type of investigation. As the costs of sequencing plummet and techniques become more refined, it is likely that whole genome sequencing technology will be used more frequently in genomic projects but also more generally in disease-based research. The vision for 2020 is that there may be considerable advances in knowledge that will enable genomic discoveries to be translated into clinical outcomes or what is becoming known as genomic medicine. However at the present time, this is not the case.^100^ In this scenario, whole genome sequencing will be a routine part of clinical care,^101^ sequence information being easily accessible to doctors and a component part of an individual's medical record. This means that questions about access to an individual's personal genome will become even more pertinent and the compelling moral arguments that an individual should have access to their personal genome will become more difficult to rebut.

Within the commercial sector, direct-to-consumer testing companies are already offering results based on genome sequencing^102^ and starting to develop the practices and know-how that will enable them to integrate whole genome sequencing into the services that they currently provide to consumers. There is the increasing likelihood that individuals in society can and do access information about their genome through commercial providers. Whilst researchers may rest assured that they will not be faced with a deluge of successful claims to access to information from research, on the other hand, medical practitioners are likely to be faced with an ever increasing number of patients wishing to have information about their genome interpreted and used to inform medical treatment. Thus, even in the absence of the right of access discussed in this paper, we feel it is essential that the implications of the growth of genomic information for the healthcare system be addressed and the way that this is changing the nature of the relationship between research participants and researchers. Appropriate policies need to be developed to deal with the greater use of whole genome sequencing, and how this information might be accessed by the individuals who provide it, not just to inform research but also to inform an individual's healthcare decision-making.

## FUNDING

J.K. Wellcome Trust Award 096599/2/11/Z; H.G. CDGG11a ESRC 07-08; K.M. is supported by the National Institute for Health Research (NIHR) Oxford Biomedical Research Centre.

## Footnotes

1 FS Collins, M Morgan, and A Patrinos, ‘The Human Genome Project: Lessons from Large-Scale Biology’ (2003) 300 Science 286–90.

2 See the special issue of Nature (2001) 409, 745–964*.*

3 National Human Genome Research Institute, ‘Human Genome Project Completion: Frequently Asked Questions’ (*genome.gov*) <http://www.genome.gov/11006943> accessed 12 March 2013.

4 <http://genomics.xprize.org/> accessed 12 March 2013.

5 SF Kingsmore, ‘CJ Saunders, Deep Sequencing of Patient Genomes for Disease Diagnosis: When Will It Become Routine?’  (2011) Sci Transl Med 3, 87ps23.

6 D Marks and others, ‘A Review on the Diagnosis, Natural History, and Treatment of Familial Hypercholesterolaemia’ (2003) 168(1) Atherosclerosis 1–14.

7 M Gymrek and others, ‘Identifying Personal Genomes by Surname Inference’ (2013) 339 Science 321–24; LL Rodriguez and others, ‘The Complexities of Genomic Identifiability’ (2013) 339 Science 275–6.

8 S Wolf and others, ‘Managing Incidental Findings in Human Subjects Research: Analysis and Recommendations’ (2008) 36 J Law Med Ethics 219.

9 RC Green and others, ‘ACMG Recommendations for Reporting of Incidental Findings in Clinical Exome and Genome Sequencing’ (2013) Genet Med doi:10.1038/gim.2013.73.

10 For some examples, see 23andMe <https://www.23andme.com/> and Navigenics <http://www.navigenics.com/> accessed 30 July 2013.

11 See, for example, Susan M Wolf, ‘The Past, Present, and Future of the Debate over Return of Research Results and Incidental Findings’ (2012) 14 Genet Med 355–7; BM Knoppers and others, ‘The Emergence of an Ethical Duty to Disclose Genetic Research Results: International Perspectives’ (2006) 14 Eur J Hum Genet 1170–8; V Ravitsky and BS Wilfond, ‘Disclosing Individual Genetic Results to Research Participants’ (2006) 6(6) Am J Bioeth 8–17; EW Clayton and others, ‘Managing Incidental Genomic Findings: Legal Obligations of Clinicians’ [2013] Genet Med doi:10.1038/gim.2013.7.

12 See n 8 above.

13 Directive 95/46/EC of the European Parliament and of the Council of 24 October 1995 on the protection of individuals with regard to the processing of personal data and on the free movement of such data.

14 Council of Europe Convention for the Protection of Human Rights and Dignity of the Human Being with regard to the Application of Biology and Medicine: Convention on Human Rights and Biomedicine. Oviedo, 4.IV.1997.

15 UNESCO Declaration on the Human Genome and Human Rights 1997.

16 Under the Human Tissue Act 2004 s 45 it is possible to get access to a sample for DNA testing without consent if it is approved by the HTA; A Lucassen and J Kaye, ‘Genetic Testing without Consent: The Implications of the New Human Tissue Act 2004’ (2006) 32 J Med Ethics 690 doi:10.1136/jme.2005.013631. However, for the purposes of this paper, we are focusing on sequence information and therefore will not discuss the law concerning samples and access by individuals to samples.

17 CA Cassa and others, ‘My Sister's Keeper?: Genomic Research and the Identifiability of Siblings’ (2008) 1 BMC Med Genomics 32.

18 LM Beskow, ‘Considering the Nature of Individual Research Results’ (2006) 6(6) Am J Bioeth 38; KA Quaid, NM Jessup, and EM Meslin, ‘Disclosure of Genetic Information Obtained Through Research’ (2004) 8 Genet Test 347; V Ravitsky and BS Wilfond, ‘Disclosing Individual Genetic Results to Research Participants’ (2008) 6 (6) Am J Bioeth 8; D Wendler and E Emanuel, ‘The Debate Over Research on Stored Biological Samples: What Do Sources Think? (2002) 162 Arch Intern Med 1457.

19 J Gitschier, ‘Inferential Genotyping of Y Chromosomes in Latter-Day Saints Founders and Comparison to Utah Samples in the HapMap Project’ (2009) 84 Am J Hum Genet 251.

20 See for example Z Lin, AB Owen, and RB Altman, ‘Genetics. Genomic Research and Human Subject Privacy’ (2004) 205 Science 183, discussed in C Heeney and others, ‘Assessing the Privacy Risks of Data Sharing in Genomics’ (2011) 14 (1) Public Health Genomics 17.

21 N Homer and others, ‘Resolving Individuals Contributing Trace Amounts of DNA to Highly Complex Mixtures using High-density SNP Genotyping Microarrays’ (2008) 4(8) PLoS Genet e1000167 <http://www.plosgenetics.org/article/info%3Adoi%2F10.1371%2Fjournal.pgen.1000167> accessed 12 March 2013.

22 See for example the plan for 100,000 NHS to have their whole genome sequenced: <http://www.number10.gov.uk/news/dna-tests-to-fight-cancer/> accessed 30 July 2013.

23 We recognise that the HTA might provide a means for a patient to obtain access to a physical sample, from which they might obtain the genomic information, thus circumventing the law regulating access to information. However, that patient would also be able to provide an additional sample from which DNA might be obtained.

24 Legally, patients have an almost absolute right to access their medical record. This is not a common law right (*R v Mid Glamorgan FHSA ex p Martin* [1995] 1 All ER 356) but is guaranteed through various statutory rights contained within the Supreme Court Act 1981 (for discovery of medical records in the context of litigation), the Access to Medical Reports Act 1988, Access to Health Records Act 1990, and the Data Protection Act 1998. The NHS Constitution also provides for a right of access to a person's own health records, although this document is without any binding legal force.

25 Direct-to-consumer genetic testing companies such as 23andMe or Navigenics are increasingly providing this raw information to their customers. 23andMe provides access to raw data for some customers <https://www.23andme.com/you/faqwin/usinggenomeexplorer> accessed 12 March 2013.

26 BC Venter and J Watson chose to make their whole genome sequence public in 2007–08; S Levy and others, ‘The Diploid Genome Sequence of an Individual Human’ (2007) 5 (10) PLoS Biol e254 <http://www.plosbiology.org/article/info:doi/10.1371/journal.pbio.0050254#aff3> accessed 12 March 2013; DA Wheeler and others, ‘The Complete Genome of an Individual by Massively Parallel DNA Sequencing’ (2008) 452 Nature 872.

27 These base pairs are AGCT and make up the human genome.

28 See for example SNPedia <http://www.snpedia.com/index.php/SNPedia> and the associated Promethease <http://www.snpedia.com/index.php/Promethease> accessed 30 July 2013.

29 UK10K Ethical Governance Framework <http://www.uk10k.org/ethics.html> accessed 12 March 2013.

30 See n 8 above.

31 We recognise that much of this information may also have some health or reproductive significance, and thus the extent to which such findings can be clearly separated from healthcare issues is unclear. However, we feel that there will be cases where it is difficult or impossible to demonstrate healthcare relevance, and it is therefore important to include these types of findings in our discussions for this paper.

32 The Nuremberg code, which followed on from the Nuremberg trials, established the principle of voluntary, informed consent for research. *Trials of War Criminals before the Nuremberg Military Tribunals under Control Council Law No. 10*, (Vol. 2, US Government Printing Office,1949) Art 1 at pp 181–2. The requirement of informed consent is further developed and enshrined in principle 24 of the World Medical Association, ‘Declaration of Helsinki—Ethical Principles for Medical Research Involving Human Subjects’.

33 T Beauchamp and J Childress, *Principles of Biomedical Ethics* (Oxford University Press, Oxford 1979).

34 This literature is considerable. A symposium in the American Journal of Bioethics, 6:6, 2006, provides an early indication of the move toward considering the obligations to feed back findings, which extends by 2013 to consideration of obligations actively to search for them. See C Gliwa and B Berkman, ‘Do Researchers Have an Obligation to Actively Look for Genetic Incidental Findings?’ (2013 ) 13 Am J Bioeth 2 and peer responses.

35 AL Bredenoord and others (Disclosure of Individual Genetic Data to Research Participants: the Debate Reconsidered (2011) 27(2) Trends Genet 41–47) summarise that ‘[r]esearch results can be disclosed passively or actively’ and that ‘[p]assive disclosure refers to disclosure on explicit request by a research participant’. They further state that ‘[t]he right to have access to one's personal, genetic, and medical data is recognized in many international and national legal guidelines’. In support, the authors chiefly cite the CIOMS International Ethical Guidelines for Biomedical Research Involving Human Subjects (2002) and the Council for International CIOMS (Organizations of Medical Sciences) International Ethical Guidelines for Epidemiological Studies (2007). However, they immediately add that ‘[t]he central discussion in this respect is not whether participants can access their data, but whether researchers should actively offer genetic results to individual research participants’. Indeed, the CIOMS 2002 Guidelines state, under Guideline 5 relating to Obtaining informed consent: Essential information for prospective research subjects, under point 8 stipulate, that: ‘subjects have the right of access to their data on demand, even if these data lack immediate clinical utility (unless the ethical review committee has approved temporary or permanent non-disclosure of data, in which case the subject should be informed of, and given, the reasons for such non-disclosure’. The same wording is used verbatim in the 2007 Guidelines as well. With the exception of these two documents—in essence, one document—we have found no other mention of an active individual claim for access to research findings in previous or subsequent documents.

36 J Murphy and others, ‘Public Expectations for Return of Results from Large-cohort Genetic Research’ (2008) 8(11) Am J Bioeth 36; offers a comprehensive literature overview of the various arguments in favour of, and against disclosure.

37 Am J Bioeth 13:2, 2013; C Gliwa and B Berkman, ‘Response to Open Peer Commentaries on ‘Do Researchers Have an Obligation to Actively Look for Genetic Incidental Findings?’ (2013) 13 Am J Bioeth 5.

38 Ibid; also G Renegar and others, ‘Returning Genetic Research Results to Individuals: Points to Consider’ (2006) 20(1) Bioethics 24.

39 *The Complainant v Queen's University Belfast* (FS50163282) (2010) illustrates the exemptions relied upon by a public authority to refuse disclosure of information on such grounds, albeit it unsuccessfully; under s12 FOIA, a request for information disclosure may be refused on the basis of costs. Regulation 12(4)(b) of the EIR permits a public authority to refuse disclosure should the request be manifestly unreasonable, *ie* where unreasonable costs would be incurred and where there would be an unreasonable diversion of resources.

40 McGuire and others, ‘Research Ethics and the Challenge of Whole-genome Sequencing’ (2008) 9(2) Nat Rev Genet 152.

41 R Tutton and B Prainsack, ‘Enterprising or Altruistic Selves? Making up Research Subjects in Genetics Research’ (2011) 33 Soc Health Illness 1081.

42 Nuffield Council on Bioethics, ‘Medical Profiling and Online Medicine: The Ethics of ‘Personalised Healthcare’ in a Consumer Age’ (Nuffield Council on Bioethics 2010) <http://www.nuffieldbioethics.org/personalised-healthcare-0> accessed 12 March 2013.

43 B Godard, J Marshall, and C Laberge, ‘Community Engagement in Genetic Research: Results of the First Public Consultation for the Quebec CARTaGENE Project’ (2007) 10(3) Commun Genet 147; K Hoeyer, ‘Donors Perceptions of Consent to and Feedback from Biobank Research: Time to Acknowledge Diversity?’ (2010) 13 Public Health Genomics 345; Murphy and others, n 36 above; see Wendler and Emanuel, n 18 above.

44 Beskow, n 18; Quaid and others, n 18; Ravitsky and Wilfond, n 18; Wendler and Emanuel, n 18.

45 CV Fernandez and C Weijer, ‘Obligations in Offering to Disclose Genetic Research Results’ (2008) 6(6) Am J Bioeth 44; DI Shalowitz and FG Miller, ‘Communicating the Results of Clinical Research to Participants: Attitudes, Practices, and Future Directions’ (2008) 5(5) PLoS Med e91 <http://www.plosmedicine.org/article/info:doi/10.1371/journal.pmed.0050091> accessed 12 March 2013.

46 CV Fernandez and C Weijer, ibid.

47 HT Greely, ‘The Uneasy Ethical and Legal Underpinnings of Large-scale Genomic Biobanks’ (2007) 8 Annu Rev Genomics Hum Genet 343.

48 SM Wolf, ‘Incidental Findings in Neuroscience Research: A Fundamental Challenge to the Structure of Bioethics and Heealth Law’, in J Illes, B Sahakian (eds), *Oxford Handbook of Neuroethics* (OUP, Oxford, 2011) 623–34; the author stipulates that ‘…[b]ioethics and health law must now reconstitute the traditional vision of researcher duties to bring the researcher back into relationship with the research participant…’ and that ‘…[r]esearchers are no longer free to work with their data in splendid isolation…’.

49 Murphy and others, n 36; Ravitsky and Wilfond, n 18; MA Rothstein, ‘Tiered Disclosure Options Promote the Autonomy and Well-being of Research Subjects’ (2006) 6(6) Am J Bioethic 20. Recent research trends develop ways to integrate dynamic management of consent preferences, including tiered notification preferences as part of user-interactive biobanking interfaces.

50 Council of Europe Convention for the Protection of Human Rights and Dignity of the Human Being with regard to the Application of Biology and Medicine: Convention on Human Rights and Biomedicine. Oviedo, 4.IV.1997.

51 Council of Europe, Additional Protocol to the Convention on Human Rights and Biomedicine, concerning Genetic Testing for Health Purposes. Strasbourg, 27.XI.2008.

52 Oviedo Convention, Art 10(2).

53 Additional Protocol, Art 16(2).

54 ‘The NHS Constitution, the NHS belongs to us all’, (8 March 2010): ‘You have the right of access to your own health records. These will always be used to manage your treatment in your best interests’ at page 8 <http://www.nhs.uk/choiceintheNHS/Rightsandpledges/NHSConstitution/Pages/Overview.aspx> accessed 12 March 2013.

55 Access to Health Records Act 1990; Access to Medical Reports Act 1988.

56 The Data Protection Act implements the EU Data Protection Directive 95/46/EC which relates to the protection of individuals with regard to the processing of personal data and the free movement of such data.

57 Schedule 1, Part I, 6.

58 S 33(4)(a) and (b).

59 The Guide to Data Protection: <http://www.ico.gov.uk/upload/documents/library/data_protection/practical_application/the_guide_to_data_protection.pdf> accessed 30 July 2013.

60 Proposal for a Regulation of the European Parliament and of the Council on the protection of individuals with regard to the processing of personal data and on the free movement of such data (General Data Protection Regulation) 2012/0011, Released 25/1/2012.

61 [2008] UKHL 47.

62 Although this concerned The Freedom of Information (Scotland) Act, the point should apply to the FOIA.

63 It is interesting to consider that, if the data are sufficiently anonymised, and a participant successfully uses the FOIA to gain access to their data, the data will have to be personalised to identify which data are the participant's, and then the DPA will apply.

64 *Department of Health v Information Commissioner* [2011] EWHC 1430 (Admin).

65 S 41.

66 S 43.

67 However, the breach of confidence exemption is subject to a public interest test. For example, if the request for disclosure of information is made by a third party who is a relative of the data subject, and the information reveals that the relative is at a high risk of suffering *serious and preventable physical harm* as a result of a condition that he or she could be unaware of, in principle, the leading case on disclosure in the public interest (*W v Egdell* [1990] Ch 395) would apply, and disclosure should be permitted. For general discussion, see D Bell and B Bennett, ‘Genetic Secrets and the Family’ [2001] Med L Rev 130; P Case, ‘Confidence Matters: The Rise and Fall of Informational Autonomy in Medical Law’ (2003) 11 Med L Rev 208; R Gilbar, ‘Medical Confidentiality within the Family’ (2004) 18 IJLPF 195; L Skene, ‘Genetic Secrets and the Family’ (2001) 6 Med L Rev 1; S Liao, ‘Is There a Duty to Share Genetic Information?’ (2009) 35 J Med E 306; L Fleck, ‘When Is My Genetic Information Your Business’ (2010) Camb Q Healthcare Ethics 27; M Taylor, ‘Health Research, Data Protection, and the Public Interest in Notification’ (2011) 19 Med L Rev 267.

68 Again, the commercial interest exemption is subject to a public interest test.

69 Although a distinction has been made between raw data and the research results reliant on this data (*The Complainant v Queen's University Belfast* (FS50163282) (2010)), this distinction was specifically made with reference to Regulation 12(4)(d) of the Environmental Information Regulations 2004 (EIR), which provides for possible exception to disclosing environmental information on the grounds of information being unfinished and incomplete.

70 See the last paragraph of the Human Rights Law section General Feedback Requests and n 7 above.

71 It is worth noting that The Freedom of Information (Scotland) Act contains an exemption for information obtained in the course of a research project, requiring substantial prejudice to the research programme, its participants, or the institution for the exemption to apply; perhaps the Information Commissioner could use the substantial detriment test as a basis for a decision as to the reasonableness of a refusal to supply information that will be published.

72 Freedom of Information Act Awareness, Guidance Number 6; <http://www.ico.gov.uk/upload/documents/library/freedom_of_information/detailed_specialist_guides/awareness_guidance_6_-_information_reasonably_accessible_to_the_applicant_by_other_means.pdf> accessed 30 July 2013.

73 Human Rights Act 1998, s 3(1).

74 Human Rights Act 1998, s 6(1).

75 Human Rights Act 1998, s 6(3)(b).

76 C Johnston and J Kaye, ‘Does the UK Biobank Have a Legal Obligation to Feedback Individual Findings to Participants?’ (2004) 12(3) Med L Rev 239.

77 See *Von Hannover v Germany* (2005) 40 EHRR 1 at [57]; and *Stubbings v United Kingdom* (1997) 23 EHRR 213 at [61]–[62].

78 Application No. 48009/08, 10 May 2011 (2011) 53 EHRR 30 at [106].

79 *Von Hannover No.2* (Case Nos 40660/08 and 60641/08) 7 February 2012 [2012] EMLR 16 at [98].

80 *Tyrer v United Kingdom*, (1979–80) 2 EHRR 1, at [31]; *Selmouni v France*, (2000) 29 EHRR 403 at [101].

81 A distinction has been drawn between such terms in various discussions about the regulation of access to genetic information, but we are not aware of any particular legal distinction, and do not seek to create one here.

82 *R (S) v Chief Constable of the South Yorkshire Police and R (Marper) v Chief Constable of the South Yorkshire Police* [2004] 1 WLR 2196.

83 *S. and Marper v The United Kingdom* (2008) 158 NLJ 1755, [2008] ECHR 1581, (2009) 48 EHRR 50, 25 BHRC 557, 48 EHRR 50. [2009] Crim LR 355 at [72].

84 *LS, R (on application of) v South Yorkshire Police (Consolidated Appeals)* [2004] UKHL 3 at [71].

85 *GC v The Commissioner of Police of the Metropolis* [2011] UKSC 21.

86 *Hendrik Jan van der Velden v the Netherlands* [2006] ECHR 1174.

87 *K.H. and others v Slovakia* App 32881/04 [2009] ECHR 709.

88 41723/06 [2010] ECHR 1676 (2 November 2010).

89 Ibid at [112]–[114] and the concurring opinion of Judge Power.

90 In the period between 1977 and 1992, a research project was carried out at the University of Gothenburg in the field of neuropsychiatry focusing on the incidences of Attention Deficit Hyperactivity Disorder (ADHD) or Deficits in Attention, Motor Control and Perception (DAMP) in children.

91 See concurring opinion of Judge Power.

92 M Spurrier, ‘Gillberg v Sweden: Towards a Right of Access to Information Under Article 10?’(2012) 5 EHRLR 551–8.

93 Under Art 9 of the draft General Data Protection Regulation however, genetic data will explicitly be classed as a special category of personal data.

94 DPA, s 33(1)(a).

95 The concern that raw data in itself may not serve any meaningful purpose when assessing its possible publication has not been considered as a valid reason for non-disclosure as stated in *The Complainant v Queen's University Belfast (FS50163282) (2010)*. It is important to note that the Information Commissioner's comments in this decision were made with respect to possible disclosure of environmental information, which falls under the scope of the EIR and not the FOIA.

96 FOIA, s 22.

97 Current practice, however, reveals that there is a high trend where scientists do not in fact publish raw data. AA Alsheikh-Ali and others ‘Public Availability of Published Research Data in High-Impact Journals’ (2011) 6 (9) PLoS ONE e24357 doi:10.1371/journal.pone.0024357.

98 See n 9 above.

99 For example the 1000Genomes Project <http://www.1000genomes.org/> and the UK10K Project <http://www.uk10k.org/> accessed 30 July 2013.

100 EDGreen, MS Guyer, and the National Genome Research Institute, ‘Charting a Course for Genomic Medicine from Base Pairs to Bedside’ (2011) 470 Nature 204.

101 Both these current and prospective advances have consequently called for greater regulation in a bid to better protect individuals with regards to the processing of personal data. The EU draft General Data Protection Regulation, published in January 2012, reflects the intended increase of individuals to control their data—European Commission Proposal for a Regulation of the European Parliament and of the Council on the protection of individuals with regard to the processing of personal data and on the free movement of such data (General Data Protection Regulation) COM(2012) 11 final, Brussels, 25 January 2012.

102 This is not whole genome sequencing but only partial as it focuses on specific single-nucleotide polymorphisms on the genome rather than all of the bases pairs in the genome.

